# Bisphenol A Removal by the Fungus *Myrothecium roridum*IM 6482—Analysis of the Cellular and Subcellular Level

**DOI:** 10.3390/ijms221910676

**Published:** 2021-10-01

**Authors:** Anna Jasińska, Adrian Soboń, Sylwia Różalska, Paulina Średnicka

**Affiliations:** 1 Department of Industrial Microbiology and Biotechnology, Faculty of Biology and Environmental Protection, University of Łódź, 12/16 Banacha Street, 90-237 Łódź, Poland; sylwia.rozalska@biol.uni.lodz.pl; 2 LabExperts, 14 Sokola Street, 93-519 Łódź, Poland; adrian.sobon@labexperts.com.pl; 3 Laboratory of Biotechnology and Molecular Engineering, Department of Microbiology, Prof. Wacław Dąbrowski Institute of Agricultural and Food Biotechnology–State Research Institute, 36 Rakowiecka Street, 02-532 Warsaw, Poland; paulina.grzelak@ibprs.pl

**Keywords:** BPA degradation, laccase, oxidative stress, *Myrothecium roridum*, estrogenic activity reduction

## Abstract

Bisphenol (BPA) is a key ingredient in the production of epoxy resins and some types of plastics, which can be released into the environment and alter the endocrine systems of wildlife and humans. In this study, the ability of the fungus *M. roridum*IM 6482 to BPA elimination was investigated. LC-MS/MS analysis showed almost complete removal of BPA from the growth medium within 72 h of culturing. Products of BPA biotransformation were identified, and their estrogenic activity was found to be lower than that of the parent compound. Extracellular laccase activity was identified as the main mechanism of BPA elimination. It was observed that BPA induced oxidative stress in fungal cells manifested as the enhancement in ROS production, membranes permeability and lipids peroxidation. These oxidative stress markers were reduced after BPA biodegradation (72 h of culturing). Intracellular proteome analyses performed using 2-D electrophoresis and MALDI-TOF/TOF technique allowed identifying 69 proteins in a sample obtained from the BPA containing culture. There were mainly structural and regulator proteins but also oxidoreductive and antioxidative agents, such as superoxide dismutase and catalase. The obtained results broaden the knowledge on BPA elimination by microscopic fungi and may contribute to the development of BPA biodegradation methods.

## 1. Introduction

Bisphenol A (BPA) is an organic compound widely used in industry as a substrate for the production of polycarbonates (PCs), epoxy resins and other polymeric materials [1,2]. Polymers obtained from BPA are characterized by high stability, good mechanical properties and low absorption of moisture. They are used as protective coatings lining pipelines, food and drink cans and cartons, as well as for the production of CDs, DVDs, electronic and electrical equipment. Additionally, BPA is widely applied in the production of thermal paper used among others at cash registers and payment terminals [3,4]. In 2015, worldwide consumption of BPA was 7.7 million metric tons, and by 2022, it is expected to reach 10.6 million metric tons. The largest market for BPA is Asia-Pacific (with 53% of the market share), whereas 36% goes to Western Europe and the USA [5].

BPA can get into food and beverages stored in containers made of it. The factors that increase the release of BPA are low pH, elevated temperature, repeated use of containers and the chemical properties of stored substances [6,7]. As a result, it is detected in the human urine, serum of pregnant women, amniotic fluid, breast milk, umbilical cord blood and other body fluids [8]. BPA belongs to endocrine disruptors (EDCs) and interferes with the normal functioning of the endocrine system. Its action is associated with fertility disorders [9], miscarriages [10], higher risk of the development of breast, uterine and ovarian cancer [11,12]. It may also cause increased susceptibility to the development of metabolic diseases such as insulin resistance, obesity and type II diabetes, as well as thyroid dysfunctions [13,14].

Potential adverse health effects have led to gradual restrictions on BPA use and imports of selected BPA-containing products. Canada, the USA, and the European Union prohibited the use of BPA in the manufacture of polycarbonate feeding bottles for infants [15,16]. The xenobiotic was restricted as a substance on its own and in mixtures intended for consumer use in the EU in 2018. Additionally, its use in thermal paper has been limited. Since January 2020, thermal paper with 0.02% or more of BPA by weight cannot be placed on the EU market [17].

Main physicochemical methods of BPA elimination comprise oxidation, adsorption and membrane filtration [18,19,20]. Each removal option has its own limitations in removing BPA. These include high operational cost, generation of products more toxic than the parent compound, the necessity of regeneration of activated carbon and the low BPA removal efficiency by the saturated membranes. Microorganisms are supposed to play an important part in the process of BPA transformation [21,22,23]. Therefore, studying the possibility of using microorganisms to remove this compound from a contaminated environment is a valid approach. An interesting object of research is, in particular, microorganisms isolated from contaminated environments (soil, sewage, sediments). They often have adaptive mechanisms thanks to which they are able to break down toxic pollutants.

In order to develop an efficient method of elimination of toxic compounds, it is necessary to understand how microbial cells react in the presence of a particular compound and what happens to them during the xenobiotic transformation. This process might involve a variety of changes in specific functional, regulatory and structural proteins. Many toxic compounds may also disrupt the functioning of cellular structures, e.g., by inducing oxidative stress in microbial cells. Therefore, the investigation of microorganisms at the cellular and subcellular level could help to understand the mechanism of their metabolism and response to toxic compounds, which might contribute to the development of an efficient method of eliminating xenobiotics from the contaminated environment.

The present work demonstrates a response to BPA of the ascomycetous fungus *M. roridum* IM 6482, previously described as an effective dye degrader and laccase producer [24,25,26]. The fungus was isolated from a postindustrial area and successfully used for the elimination of dye. This suggests that adaptation to toxic compounds may be an important factor influencing biodegradation efficiency. The present study involved examination of the fungal growth and BPA elimination curve, liquid chromatography with tandem mass spectrometry (LC-MS/MS) identification of BPA metabolites formed as a result of the fungus activity and investigation of the mechanisms involved in BPA transformation. Reactive oxygen species (ROS) production, membrane permeability and lipid peroxidation were also examined to indicate subcellular changes during BPA biotransformation. Proteomic tools were applied for the analysis of changes in intracellular proteins in the presence of BPA. A yeast estrogen bioassay was performed to check the estrogenic activity of BPA and its metabolites. To the best of our knowledge, this is the first work presenting such a comprehensive investigation of fungal cell response to BPA presence and its fate during the transformation by the *M. roridum* strain.

## 2. Results and Discussion

### 2.1. Fungal Growth, BPA Biodegradation and Laccase Involvement

**Figure 1:**
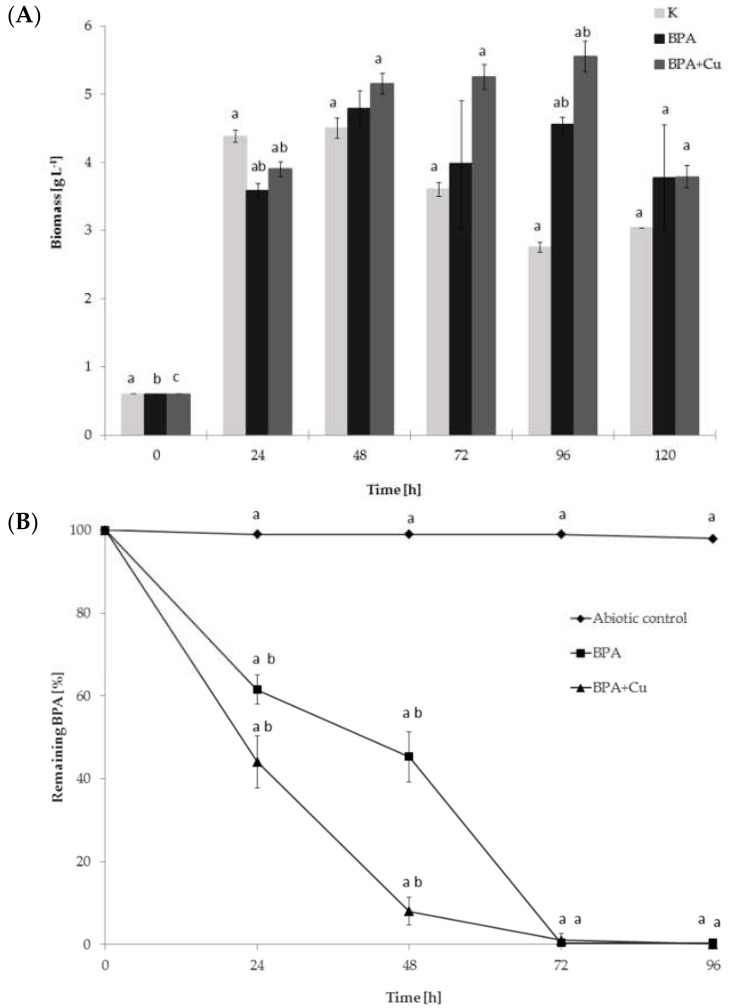
(**A**) The biomass and (**B**) kinetics of BPA elimination in *M. roridum* cultures incubated with the addition of BPA at a concentration of 50 mg·L^−1^ on Czapek-Dox III medium. Letters represent values with statistically significant differences determined with a one-way ANOVA followed by the Tukey test (α = 0.05) for different treatments.

The growth and degradability of BPA by the ascomycetous fungus *M. roridum* IM 6482 was assessed in Czapek-Dox cultures containing 50 mg·L^−1^ of BPA. The *M. roridum* strain had been previously described as a producer of copper-induced laccase capable of decolorization of dyes [24,25,26]. Therefore, fungal growth and biodegradation of BPA by *M. roridum* IM 6482 was also investigated in the presence of copper, which is a commonly known laccase inducer. The addition of BPA or BPA with CuSO_4_ slightly limited the growth of the fungus on the first day of cultivation (Figure 1A). However, after 48 h, the cultures containing these compounds showed a significant increase in the biomass content compared to the cultures containing neither BPA nor copper ions. The highest intensification of biomass production was observed after 96 h of incubation in the cultures containing BPA and Cu^2+^. The biomass content in this system was more than half higher than in the control system and was equal to 5.55 g·L^−1^. It may suggest that BPA, which initially had been toxic to fungal cells, was probably then transformed and used as the source of carbon and energy by *M. roridum* IM 6482. BPA itself could not serve as the primary energy source for those organisms (data not shown), which indicates that transformation of BPA by *M. roridum* IM 6482 takes place through co-metabolism. This mechanism of xenobiotic degradation is common in fungi and involves the transformation of a non-growth substrate in the obligate presence of a growth substrate or another transformable compound [27,28].

The biodegradability of BPA has been demonstrated in many species of fungi and bacteria [29,30,31]. The elimination of this compound with the participation of laccase produced by white rot fungi is also well characterized [32,33]. However, relatively little work is devoted to BPA biodegradation by ascomycete fungi growing under the conditions of laccase induction. Mtibaà et al. [34] described four ascomycete fungi (one *Chaetomium* and three *Thielavia* strains) as capable of BPA removal. *C. strumarium* G5I was found to be the most efficient degrader, showing 100% of removal within 8 h of incubation. In the present study, LC-MS/MS analysis showed that after 24 h of incubation, BPA removal from the cultures grown without the copper addition achieved 38.5%, while in the cultures containing CuSO_4_, almost 60% of the xenobiotic was eliminated (Figure 1B). It suggests that in the copper-containing cultures, there was a factor, probably extracellular laccase, which could have increased BPA elimination. The extension of the culturing time to 48 h resulted in an increase in BPA removal from the culture containing BPA or BPA with Cu^2+^ to 54.7 and 92%, respectively. After 72 h, BPA was transformed almost completely in both culture variants.

**Figure 2:**
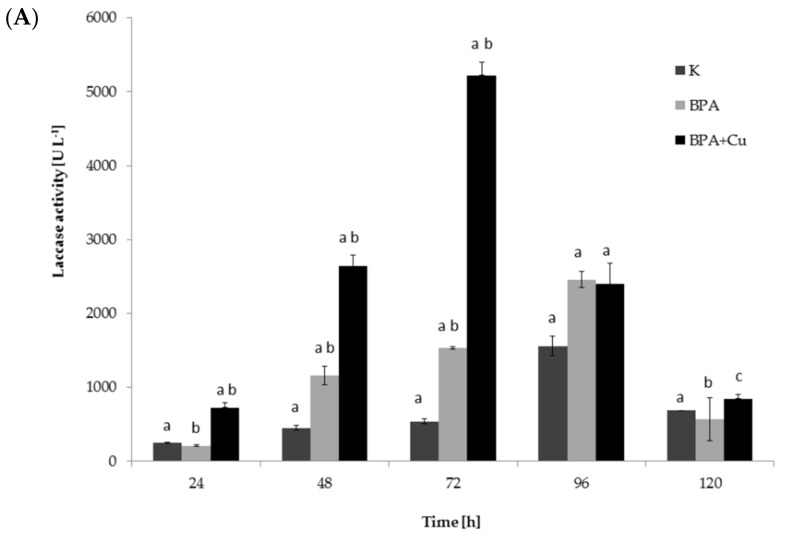

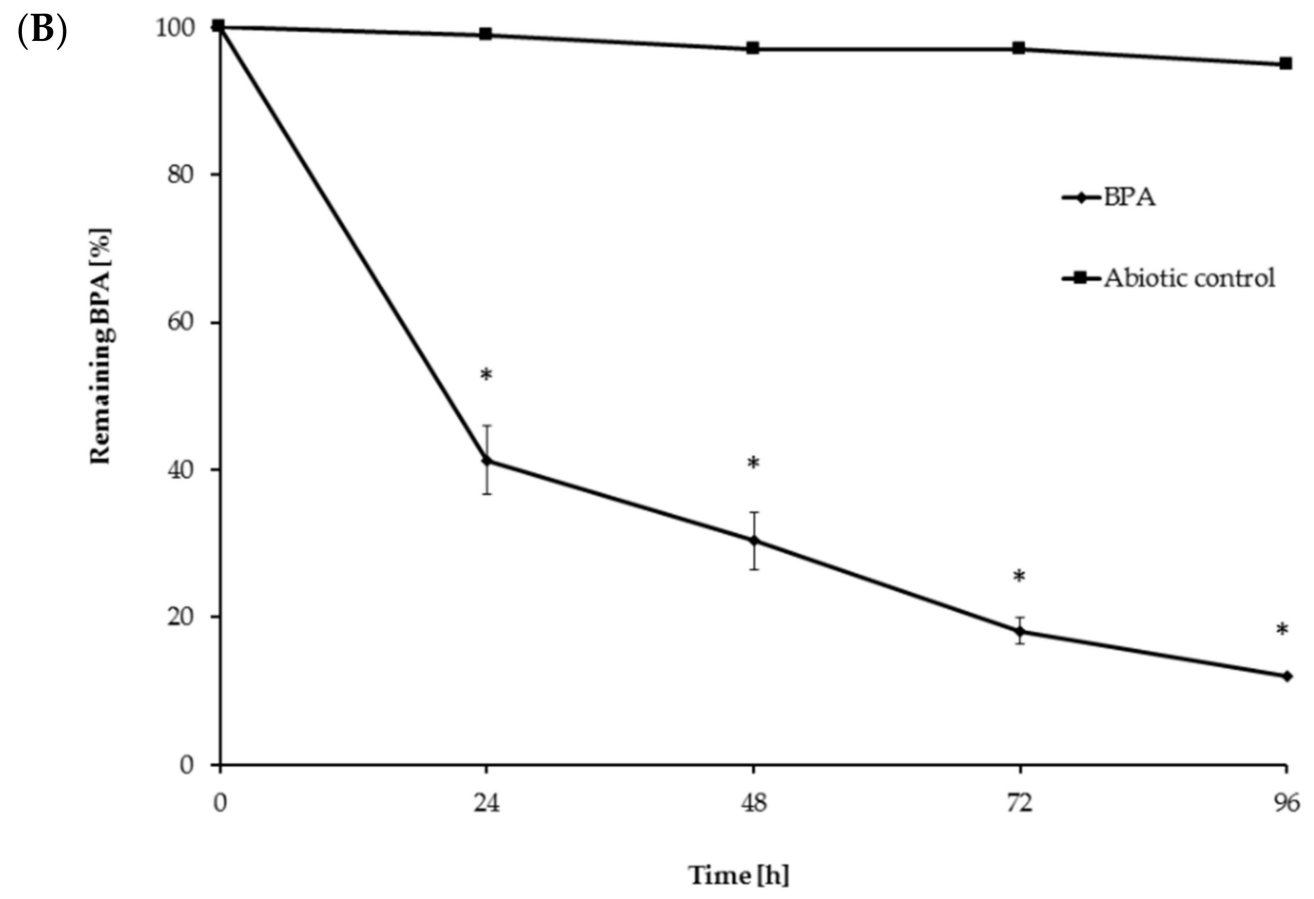
(**A**) Laccase activity in *M. roridum* cultures cultivated in Czapek-Dox III medium with BPA (50 mg·L^−1^), BPA (50 mg·L^−1^) and copper sulfate (1 mM) or without BPA supplementation. (**B**) BPA removal from water solution by crude laccase of *M. roridum* (1 U·mL^−1^). Letters and asterisks represent values with statistically significant differences determined with a one-way ANOVA followed by the Tukey test (α = 0.05) for different treatments.

The activity of laccase was assessed in the cultures containing BPA or BPA with copper ions and compared to the control cultures without these compounds. Increased production of the enzyme was observed in the cultures containing BPA, as well as in the cultures grown in the presence of Cu^2+^ and BPA (Figure 2A). The highest enzyme activity (5100 U·L^−1^) was determined in the samples after the simultaneous addition of BPA and Cu^2+^ ions, and it was three times higher than in the systems containing only BPA.

Additionally, crude laccase isolated from 48-h *M. roridum* cultures was used to eliminate BPA (50 mg·L^−1^) from aqueous solutions. The obtained results are shown in Figure 2B. After 24 h of incubation, as much as 60% BPA removal was observed. This value was similar to the elimination rate found in the cultures containing BPA and copper ions (Figure 1B). Increasing the incubation time to 72 h resulted in the elimination of over 80% BPA from the solution. The determined value was only 20% lower than the rate of the xenobiotic elimination from the *M. roridum* culture. It can be supposed that xenobiotics degradation mediated by laccase may be improved by the addition of small molecular redox mediators. For example, BPA oxidation by laccase in the presence of redox mediators, such as acetosyringone, 1-hydroxybenzotriazole (HBT), 2,2,6,6-tetramethylpiperidine-1-oxyl (TEMPO) and 2,2′-azino-bis(3-ethylbenzothiazoline-6-sulfonic acid) (ABTS), has been described previously [35,36,37,38]. However, this significantly increases operation costs and can generate secondary contaminants. Crude laccase extracts (compared with purified enzymes) often contain natural mediators, which promote BPA removal and decrease the costs of the process [32]. Therefore, the approach applied in the presented work, using raw laccase extract to eliminate BPA, seems to be an economic, fast and low-cost alternative to other methods used in the elimination of xenobiotics.

### 2.2. Identification of the Intermediates of BPA Transformation

For the determination of the products of BPA transformation by *M. roridum* IM 6482, fungal cultures containing BPA (50 mg·L^−1^) and BPA with CuSO_4_ (1 mM) were prepared. Extraction with the QuEChERS method was followed by chromatographic analysis. A qualitative trend analysis of the remaining compounds by comparing the extracted ion peak areas was also conducted. Table 1 presents BPA intermediates identified in the current work. Peak intensity analysis showed that after 24 and 48 h of cultivation predominated mainly derivatives marked as M1, M2, M3 with *m*/*z*: 259, 307 and 453 (two different BPA dimers) and retention times 8.46, 8.16 and 11.31/11.80, respectively. BPA dimers were present in small amounts in 24 and 48 h of the culture, but in 72 and 96 h, their number increased significantly. Additionally, BPA-G glucuronide conjugate (M4) with *m*/*z* 415 and retention time 12.932 was identified. In addition, in the cultures containing copper ions, a derivative (M5) with a retention time of 8.846 and with *m*/*z* 243 was detected. That metabolite was not present in the culture without copper supplementation. After BPA treatment with crude laccase, only the presence of BPA dimers was observed. Dimers were detected after 24 h of incubation. In the following hours of the culture, their level gradually decreased. Mass spectra of all identified compounds are presented in the Supplementary Materials (Figure S1). These results are consistent with earlier findings, which indicate dimers and trimers as products of BPA oxidation via laccase [39,40,41]. BPA oligomers can then be converted to low molecular weight derivatives, e.g., 4-isopropenylphenol.

### 2.3. Analysis of Cellular Response to BPA

The toxic effect of xenobiotics may manifest itself in the cells of microorganisms not only in the form of growth limitation but also by damaging and denaturing biological molecules and changes in cell membranes [42]. Therefore, the intracellular response to the BPA presence in the growth environment was analyzed. The influence of BPA on *M. roridum* cells was established by analyzing the condition of cell membranes, inspecting the ROS presence in fungal cells as well as assessing the degree of cell lipid peroxidation. The analyses were done in 24 and 72 h of the biodegradation process. Additionally, mycelium samples collected after 48 h of culturing were applied for intracellular proteome expression studies.

#### 2.3.1. Permeability of Fungal Membrane

Cell membranes are the first line of resistance of a microorganism to toxic compounds present in the extracellular environment. Hydrophobic organic compounds can disturb membranes integrity, permeability and fluidity [43]. The changes observed at the membrane level can tell a lot about the toxicity of a compound as well as about the adaptation and defense mechanisms of a microorganism. The influence of BPA on *M. roridum* membrane permeability was assessed within the first 72 h of the biodegradation process by staining the mycelium with propidium iodide. The dye is not bound inside a healthy and undamaged cell. However, when the cell membrane is damaged and/or its permeability increases, propidium iodide penetrates inside the cell and binds to the DNA. It turned out that the presence of BPA in the growth medium significantly increased the permeability of *M. roridum* cell membranes (Table 2). For the mycelium from the 24-h culture grown with BPA, the fluorescence was about eight times higher than that of the control system (without BPA addition). Interestingly, in the case of the mycelium collected after 72 h of cultivation, this difference decreased. This may indicate the conversion of BPA to less toxic derivatives and activation of defence mechanisms. Similar results had been previously obtained for different fungi exposed to pesticides [44,45,46], heavy metals [47] and antifungal natural compounds [48,49].

#### 2.3.2. ROS Production

The toxic effect of xenobiotics on microorganisms may result from the production of ROS by their cells. For example, an increased ROS content was found during the biodegradation of alachlor by *Paecilomyces marquandii* [50], tributyltin by *Cunnighamella echinulata* [51] and alachlor and metolachlor by *Trichoderma* spp. [52]. The ROS increase induced by BPA has been reported in several cell types, and there is growing evidence that the induction of ROS by BPA may contribute significantly to its toxicity and carcinogenic potential [53]. In the present work, the results obtained after mycelium incubation with 2′,7′-dichlorodihydrofluorescein diacetate (H_2_DCFDA) showed an increased generation of ROS during the first 24 h of cultivation with BPA compared to the control culture (Table 2, Figure S2). The ROS level expressed as s a percentage of the green fluorescence area compared to the total hyphal area was over 140-fold higher in the mycelium from the cultures cultivated with BPA than from the control cultures (without the xenobiotic). Surprisingly, for biomass obtained after 72 h of culturing, this difference was reduced to 6 times. This is in line with the results obtained for membranes permeability.

#### 2.3.3. Cellular Lipids Peroxidation

ROS generated in response to toxic compounds can result in oxidative damage to cellular lipids. BPA has been proven to induce lipid peroxidation in human, animal and plant cells [54,55,56]. Therefore, the effect of BPA on the formation of thiobarbituric acid-reactive substances (TBARS), which are considered popular biomarkers of lipid peroxidation, was assessed. The results are presented in Table 2. In the BPA-treated mycelium obtained from 24-h cultures, an enhancement in the TBARS level was observed compared to the mycelium from the cultures without BPA (4.02 and 1.43, respectively). No significant differences were observed for the mycelium obtained from the 72-h culture (when BPA was almost completely removed from the growth medium). It confirmed the induction of oxidative stress in the presence of BPA.

#### 2.3.4. Proteomic Analysis

**Figure 3:**
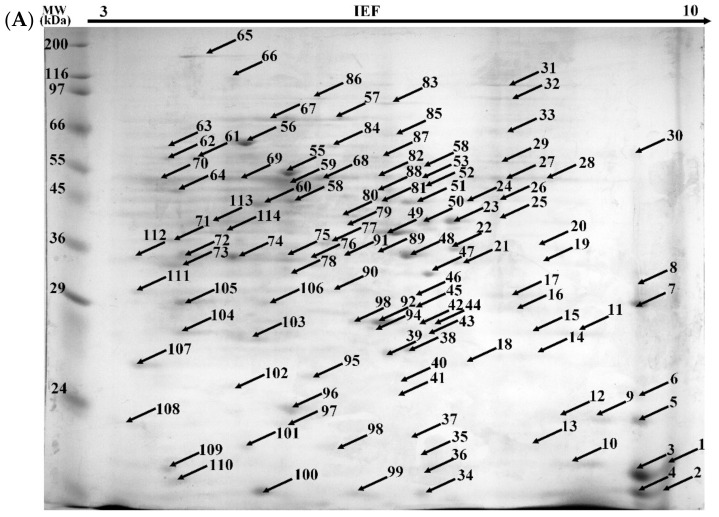

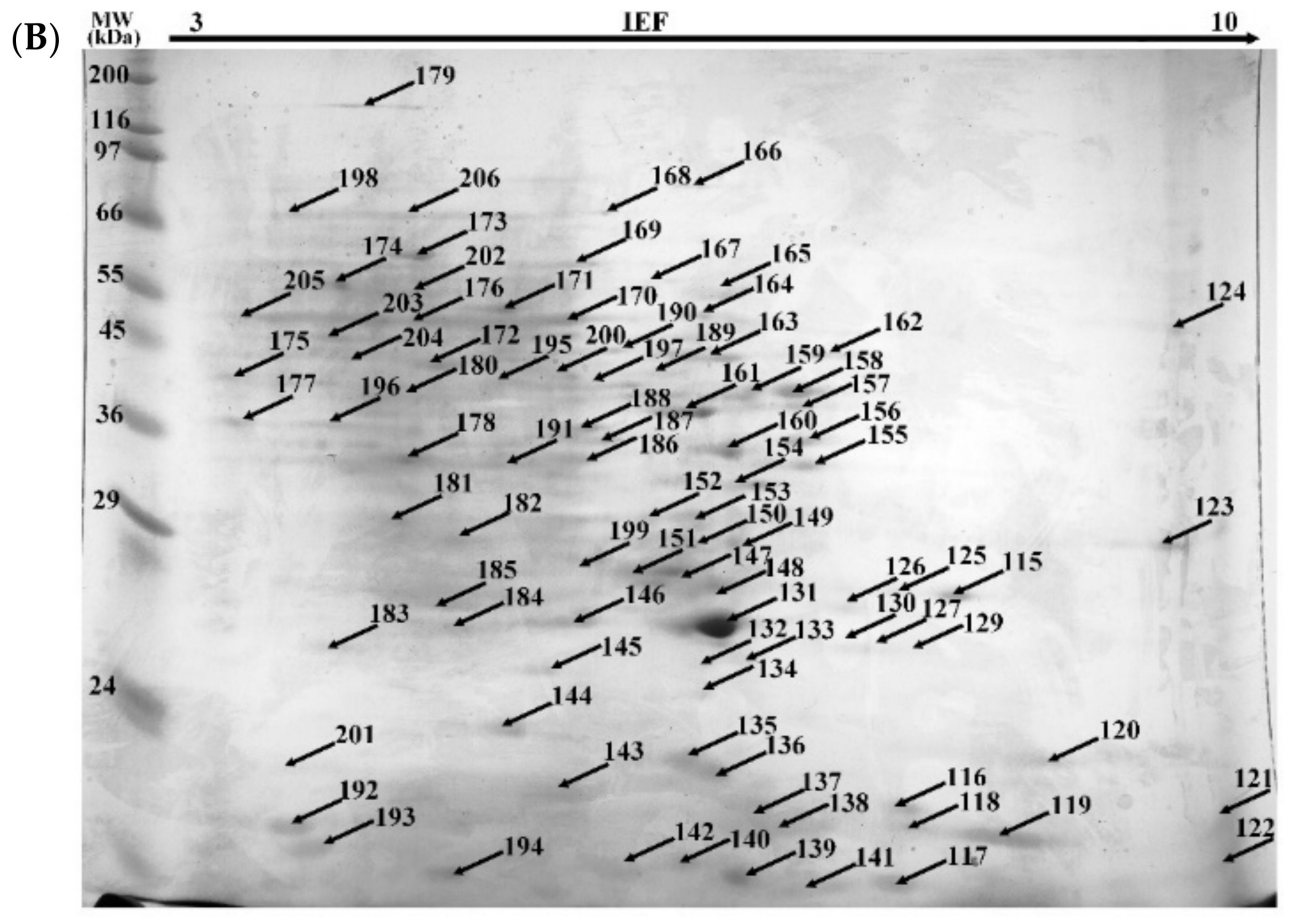
Representative 2-DE gels of intracellular proteome of *M. roridum* IM 6482 obtained from (**A**) control cultures (without BPA) and (**B**) after exposure to 50 mg·L^−1^ BPA.

The extraction of the intracellular proteome was followed by 2-D electrophoresis (2-DE) separation. In the control sample (cultures without BPA) and in the BPA-containing sample, 114 and 91 protein spots were observed, respectively (Figure 3). Among them, respectively, 40 and 69 proteins were identified (Tables S1 and S2). The presence of structural and regulatory proteins was noted in both the control and xenobiotic cultures. The presence of proteins involved in the structure and functioning of the cytoskeleton, such as profilin, calmodulin and actin was detected. The enzymes involved in basic metabolic pathways were identified: enolase, glyceraldehyde-3-phosphate dehydrogenase, glucose-6-phosphate dehydrogenase and malate dehydrogenase. The proteins involved in energy processes (ATP synthase) and ribosomal proteins (60S, 40S, 19S) were also detected. However, numerous proteins associated with the occurrence of oxidative stress (peroxyredoxins, superoxide dismutase), heat shock proteins and oxidoreductases (catalase, peroxidase) were found in the culture containing BPA. The upregulation of antioxidative enzymes, which can be represented by manganese superoxide dismutase and catalase, was observed in the alachlor- and nonylphenol-containing cultures of *M. robertsii* [50,57].

### 2.4. Estrogenic Activity of BPA and Its Biotransformation Products

**Figure 4:**
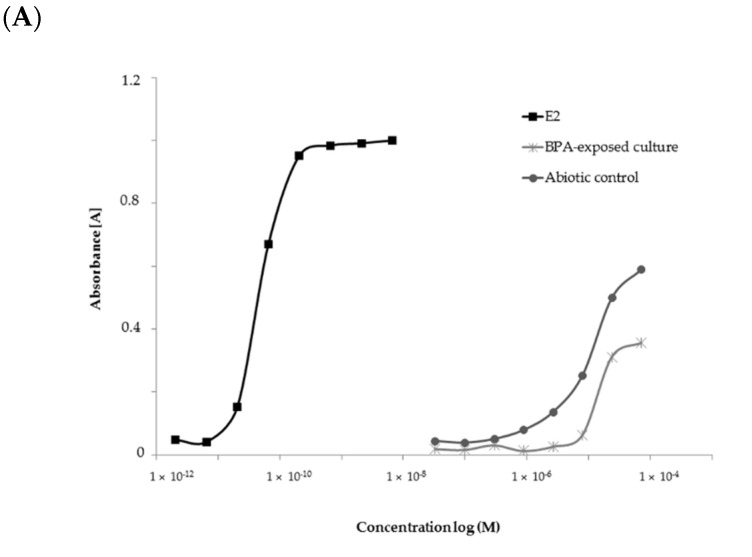

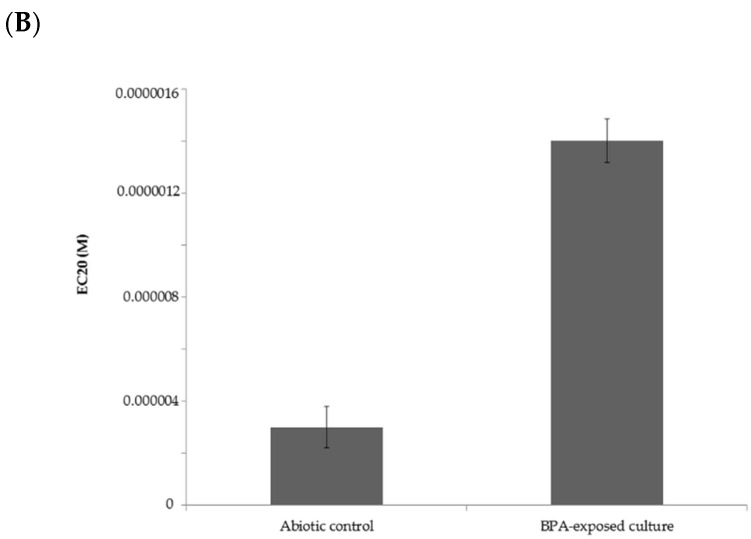
(**A**) ER dose-response curves and (**B**) EC20 (M) values for extracts of abiotic control and *M. roridum* IM 6482 culture cultivated in Czapek-Dox III medium containing the addition of BPA (50 mg·L^−1^).

The estrogenic activity of the abiotic control (containing only Czapek-Dox III medium supplemented with 50 mg·L^−1^ BPA) and extract obtained from the *M. roridum* IM 6482 culture cultivated in the presence of BPA was assessed by the yeast estrogen screen (YES) bioassay. To compare the estrogenic activity of the analyzed samples, 17β-estradiol (E2) was used as a positive control. Both tested samples showed a significantly lower estrogenic potency than native E2 (Figure 4A). This is in line with literature data. For example, Vega-Morales and Sosa-Ferrera [58] reported that the average estradiol equivalency factor value for BPA is 3.9 × 10^−4^. BPA metabolites obtained as an extract from the *M. roridum* culture exhibited significantly lower estrogenic potency than BPA alone at the tested concentration. This observation might indicate that derivatives obtained through biological transformation have lower receptor affinity or efficacy against the estrogen receptor (ER) [59].

At the tested concentrations, no inhibition of yeast cell growth was observed in any of the analyzed samples, which means that the observations were not caused by the changed yeast growth rate or cytotoxicity. Estrogenic activity was expressed as EC20 values. The results shown in Figure 4B indicate that there was an effective reduction in the estrogenic activity of BPA in the cultures of *M. roridum* IM 6482. According to the literature, -orto hydroxylated BPA metabolites are less estrogenic than BPA [60,61]. Furthermore, it is suspected that BPA dimers are characterized by a reduced estrogenic activity due to the inability to bind to the estrogen receptor because of too high molecular weight [62]. Those results are in line with the results of this study. Out of the identified metabolites, hydroxylated derivatives, glucuronides and dimers were the most abundant, which explains the lower estrogenic potency of the compounds observed in the test applied.

## 3. Materials and Methods

### 3.1. Chemicals and Microorganisms

BPA (≥98%) was purchased from Sigma-Aldrich (St. Louis, MO, USA), CHCA MALDI matrix was purchased from CovaChem (Loves Park, IL, USA). The other chemicals and reagents were obtained from POCH (Gliwice, Poland), Serva (Heidelberg, Germany), Chempur (Piekary Śląskie, Poland), Sigma-Aldrich (St. Louis, MO, USA), BioShop (Burlington, ON, Canada), Bio-Rad (Hercules, CA, USA), Thermo Fisher Scientific (Waltham, MA, USA), Promega (Fitchburg, WI, USA). All reagents were of analytical grade. Buffers and solutions were prepared in distilled water. Water using for solutions preparation used in the proteomic analysis was ultrapure purity. Stock solutions of BPA were prepared at 50 mg·mL^−1^ of ethanol.

*Myrothecium roridum*, the filamentous fungus from the collection of the Department of Industrial Microbiology and Biotechnology, University of Lodz (identification number IM 6482), was examined in this work. This strain was collected from soil around a textile dyeing factory (Zgierz, Poland) [63].

*Saccharomyces cerevisiae* yeast strain used during the yeast estrogen bioassay was purchased from Tigret (Warsaw, Poland).

### 3.2. Batch Experiments

*M. roridum* IM 6482 cultures were grown on ZT slants at 28 °C. Fully sporulated culture slants were used to inoculate 7 mL WHI3 medium. The cultivation was performed on a rotary shaker (140 rpm) for 24 h at 28 °C. The preculture was transferred to a fresh medium (ratio 1:1, *v/v*) and incubated for the next 24 h. Thereafter, Chapek-Dox III medium [24] was supplemented with only BPA (50 mg·L^−1^) or with BPA and copper sulfate (1 mM). The cultures were inoculated with 10% of preculture, incubated at 28 °C on a rotary shaker (140 rpm), and collected every 24 h of culturing. Biotic controls did not contain BPA, or copper sulfate, whereas abiotic cultures were not inoculated with the fungus.

### 3.3. Dry Weight and Laccase Activity Determination

The fungal biomass was separated from the extracellular fluid by vacuum filtration using previously weighed Whatman filter papers No. 1 (Merck, Germany). The mycelium was dried under 80 °C until constant weight. Fungal dry weight was expressed as mg·mL^−1^.

Laccase activity in extracellular fluids was assessed using the ABTS method with some modifications previously described by Góralczyk-Bińkowska et al. [64].

### 3.4. BPA Degradation Using Crude Laccase

Crude laccase enzyme was isolated from 48-h *M. roridum* IM 6482 cultures according to the method described previously [24]. Crude laccase (1 U·mL^−1^ activity toward ABTS) was added to a water solution containing BPA (50 mg·L^−1^) and incubated for 96 hat room temperature on a laboratory orbital mixer.

### 3.5. Determination of BPA and Its Metabolites Using LC-MS/MS

BPA and its metabolites were extracted using the Quick, Easy, Cheap, Effective, Rugged, and Safe (QuEChERS) method [65]. Fungal cultures were transferred into 50-mL Falcon tubes and homogenized with the glass beads (1-mm diameter) and acetonitrile (10 mL) on a mechanical disintegrator (Retsch, Ball Mill MM 400). A salt mixture (4 g of MgSO_4_, 1 g of NaCl, 1 g of C_6_H_5_NaO_7_ × 2H_2_O and 0.5 g of C_6_H_6_Na_2_O_7_ × 1.5 H_2_O) was added to the homogenate and mixed for 2 min. The samples were centrifuged, and the top layers were collected for chromatographic analysis. LC–MS/MS analysis was performed using LC: Agilent 1200 coupled with a tandem mass spectrometer AB Sciex QTRAP 3200.

For quantitative analysis, chromatographic separation was conducted on a Kinetex C18 column (2.1 mm × 50 mm; 1.7 µm) from Phenomenex. The column oven temperature was set at 40 °C. The mobile phase consisted of water (A) and methanol (B) supplemented with 5 mM ammonium formate. The injection volume was set to 25 µL. The gradient conditions were 10% B at the beginning, linearly increased to 100% B for 12 min, held for 4 min and finally returned to the initial conditions. The flow rate was 400 μL·min^−1^. The detection of BPA was conducted using an MS/MS acquisition in multiple reaction monitoring (MRM) in the negative ionization mode. The BPA concentration was calculated on the basis of a standard equation, which showed linearity in the ranges from 0 to 100 g mL^−1^ of BPA. For BPA metabolites, we applied a trend analysis of chromatographic pMRM peak areas according to Szewczyk et al. [57].

For qualitative analyses, chromatographic separation was conducted n Zorbax XDB-C18 (4.6 mm × 50 mm, 1.8 µm) (Agilent). The column temperature was set to 30 °C, and the injection volume was set to 25 µL. The optimized ESI ion source parameters were as follows: CUR: 25; IS: −4500 V; TEMP: 600 °C; GS1: 50; GS2: 60. Based on the mass spectrum of BPA, ions 133 *m*/*z* and 149 *m*/*z* were chosen as precursor ions in the Information Dependent Acquisition (IDA) method for the identification of BPA metabolites.

### 3.6. Protein Extraction

The cultures were centrifuged (6000× *g*, 15 min), and the obtained biomass was freeze-dried. The biomass was transferred to the LoBind Eppendorf tubes with glass beads, frozen in liquid nitrogen and homogenized on FastPrep24 (MP Biomedicals, Solon, OH, USA) five times for 30 s with a velocity of 4 m·s^−1^ and a 2 min break for sample cooling on ice. The proteins were dissolved in 300–400 μL of Solubilisation Buffer (SB), which contained urea (7M), thiourea (2M), 3-[(3-cholamidopropyl)dimethylammonio]-1-propanesulfonate (CHAPS) (4%) and dithiothreitol (DTT) (10 mM). Afterwards, the samples were homogenized (15 sec) and incubated in an ultrasonic cleaner (MISONIX, England, UK) (15 min). The samples were then centrifuged (15,000×*g*, 15 min, 4 °C), and the supernatant was transferred to new LoBind Eppendorf tubes. To precipitate the protein, 40% trichloroacetic acid (TCA) in a 1:1 (*v/v*) ratio was added to the supernatant, vortexed and incubated for 60 min at −20 °C. The samples were then centrifuged (15,000× *g*, 15 min, 4 °C), and the supernatant was discarded. Protein precipitate was washed with cold acetone and centrifuged again as previously. The action was repeated three times. The remaining acetone was evaporated using a concentrator. The protein precipitate was dissolved in 300–500 μL SB buffer, incubated on an ultrasonic cleaner (10 min) and vortexed. The total protein concentration in the tested samples was determined by the Bradford method with bovine serum albumin (BSA) (Sigma-Aldrich, USA) as the protein standard.

### 3.7. 2-D Electrophoresis and Protein Digestion

The protein solution was diluted using SB buffer to obtain 1000 μg·mL^−1^ for immobilized pH gradient (IPG) strip. Isoelectric focusing was performed on immobilized non-linear pH 3–11 gradient 17 cm IPG strips. IPG strips with focused proteins were reduced using DTT and followed by alkylation with iodoacetamide (IAA). The second dimension SDS-PAGE was run on a running gel with a stacking gel on top. Electrophoretic separation was carried out using 35 mA for approximately 12 h. The gels were calibrated with a molecular mass marker of 6500–200,000 Da (Sigma-Aldrich, St. Louis, MO, USA) and stained with Coomassie blue.

After electrophoresis, protein spots were cut out from the gel, transferred to LoBind Eppendorf tubes and cut into small pieces. Gel pieces were decolorized with 100 μL of 50 mM mixture of ammonium bicarbonate and acetonitrile (NH_4_HCO_3_:ACN) (50:50, *v/v*) and dehydrated with 100 μL of ACN while being vortexed for 10 min. The supernatant was discarded, and the step was repeated two times. Protein digestion was performed using trypsin solution in 25 mM NH_4_HCO_3_. The samples were incubated overnight at 37 °C. For peptide extraction, the following solutions were added to the gel fragments, incubated on a rotary shaker (140 rpm) at 28 °C for the right amount of time, and then the supernatant was collected in new Eppendorf tubes. The following solutions were used: 0.1% trifluoroacetic acid (TFA) in 2% ACN for 20 min, 0.1% TFA in 50% ACN for 60 min and 0.1% TFA in 90% ACN for 20 min. Next, the samples were evaporated in a concentrator.

### 3.8. MALDI-TOF/TOF Analysis, Protein Homology Identification

The extracted peptides were suspended in 10 μL of a 0.1% TFA solution in 2% ACN and incubated on an ultrasonic washer for 15 min. Then a 10 μL CHCA MALDI matrix was added. After vortexing, the samples were transferred to a MALDI plate and analyzed using the AB Sciex 5800 TOF/TOF system (AB Sciex, Framingham, MA USA). MASCOT and BLAST searches were performed according to Szewczyk et al. [57]. Proteins were identified by comparing the amino acid sequences of the obtained peptides with sequences contained in the NCBI database related to fungi using the Protein Pilot v 4.5 software with the MASCOT search engine v 4.5.

### 3.9. Lipid Peroxidation Assay

The degree of lipid peroxidation was measured spectrophotometrically as the content of TBARS according to the method described by Słaba et al. [47]. Fresh wet biomass (0.5 g) was homogenized with a ball mill (MM 400, Retsch) with 9 mL of deionized water and 50 μL of 7.2% thiobarbituric acid (BHT) in ethanol. Then, 1 mL of the homogenate was transferred to a Falcon tube containing 2 mL of TBA-TCA solution (15 mM thiobarbituric acidin 15% TCA). The mixture was vortexed and heated for 30 min at 95 °C. The cooled samples were centrifuged for 15 min at 2000× *g* and then incubated for 10 min at RT. The absorbance of supernatants was measured using a SPECORD 200 spectrophotometer (Analityk Jena, Jena, Germany) with the parameters: λ_ex_ = 531 nm, λ_em_ = 600 nm. The level of lipid peroxidation was expressed as μM of TBARS calculated per 1 g of wet biomass.

### 3.10. Membrane Permeability

The changes in the membrane permeability were estimated according to the method described by Siewiera et al. [65]. Briefly, 1 mL of the culture was transferred into the Eppendorf tube and centrifuged for 6 min at 6000× *g*. Then, the mycelium was suspended in 1 mL of phosphate-buffered saline (PBS) (pH 7), and 2 μL of propidium iodide solution (1 mg·mL^−1^ in H_2_O) was added and incubated for 5 min at room temperature in the dark. Next, the samples were centrifuged for 5 min at 8000× *g,* and the supernatant was removed. The mycelium was washed twice with PBS, suspended in 0.5 mL of PBS and transferred to a 24-well plate. The fluorescence intensity was measured using a FLUOstar Omega spectrofluorometer (BMG Labtech, Germany) with the parameters: λ_ex_ = 540 − 10 nm, λ_em_ = 630 − 10 nm, gain = 2000. The results were shown as a fluorescence unit per 1 mg of the fungal biomass.

### 3.11. ROS Measurement

The ROS level was measured according to the method described earlier by Słaba et al. [66]. The mycelium obtained from 24- and 72-h cultures were washed with 1 mL of 10 mM PBS (pH 7.0) and centrifuged (2000× *g*, 5 min). The mycelium was resuspended in 1 mL PBS supplemented with 40 mM H_2_DCFDA (Sigma-Aldrich, St. Louis, MO, USA) and incubated for 15 min. After being washed, the samples were mounted on microscope slides, and images were captured using a confocal laser scanning microscope (LSM510 Meta, Zeiss, Munich, Germany) combined with an Axiovert 200 M (Zeiss, Munich, Germany) inverted fluorescence microscope equipped with a Plan-Apochromat objective (63/1.25 oil). The results were expressed as a percentage of the green fluorescence area compared to the total hyphal area.

### 3.12. Yeast Estrogen Bioassay

The transgenic *Saccharomyces cerevisiae* yeast strain [67] purchased from Tigret (Warsaw, Poland) was used to assess the estrogenic activity of the tested compounds, according to the method described previously [68]. Briefly, an overnight culture of YES yeast (OD 690 nm approx.1.2) was centrifuged (1000× *g*, 10 min) and re-suspended in 10 mL of fresh growth medium. The test samples were put on a 96-well plate, mixed with 120 μL of 1% DMSO solution and inoculated with 60 μL of yeast suspension, shaken by hand and incubated overnight at 30 °C in a plastic box to keep sufficient moisture. To evaluate cultures growth, the optical density of the samples was measured at 690 nm. The cultures were terminated with 20 μL of 0.1% Triton X-100 and 30 μL of lyticase (1 mg·mL^−1^) dissolved in 0.1 M phosphate buffer (pH 7.5) containing mercaptoethanol (50 mM). The samples were incubated for 45 min at 30 °C and afterwards mixed with chlorophenol red-β-d-galactopyranoside (CPRG) solution (1.0 mg·mL^−1^). The CPRG (Sigma-Aldrich, St. Louis, MO, USA) hydrolysis rate was measured at 570 nm after 30 min of incubation at 30 °C.

Galapagos 2.0 (Plate reader) software packages were used to acquire data. The samples were assessed in terms of agonist activity against a human ER.

The results were expressed as 20% maximal effective concentration (EC20) values estimated from dose-response curves plotted for samples and the native hormone (E2).

### 3.13. Data Acquisition and Statistical Analysis

The experimental data represent the means of at least three independent experiments. Sample variability is given as a standard deviation (±SD). ANOVA followed by Tukey’s test was applied to indicate statistically significant differences. Differences at *p*< 0.05 were considered significant. Data were analyzed using Microsoft Office Excel 2013 and Statistica 13.3 software.

## 4. Conclusions

In this study, for the first time, BPA elimination by the fungus from the *Myrothecium* species has been described and characterized on the cellular and subcellular levels. The obtained results allowed drawing the following conclusions:

- *M. roridum* IM 6482 is able to completely eliminate BPA from the growth environment within 72 h of incubation;
- the main biotransformation products are: hydroxylated derivatives, glucuronides and dimmers;
- BPA does not decrease biomass production by *M. roridum* IM 6482 but induces oxidative stress in fungal cells, which is manifested by increased membrane permeability, peroxidation of lipids, overproduction of ROS and oxidative stress enzymes. Oxidative stress is eliminated after BPA degradation;
- products of BPA biodegradation by *M. roridum* IM 6482 have lower estrogenic activity compared to the parent compound.

The presented results broaden the knowledge on the elimination of BPA by microscopic fungi and may contribute to the development of methods for the biodegradation of this dangerous compound.

## Figures and Tables

**Table 1:** Metabolites identified in *M. roridum* IM 6482 cultures cultivated in modified Czapek-Dox medium with BPA or BPA/copper sulfate mixture and after *M. roridum* laccase incubation in water solution containing 50 mg*·*L^−1^ BPA.

ID	(M − H) ^−^*m*/*z*	R_t_ (min)	Structure
M1 *•	259	8.46	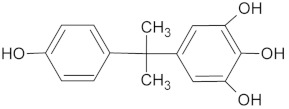
M2 *•	307	8.16	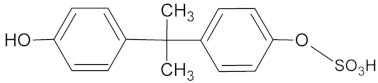
M3 *•#	453	11.31 11.80	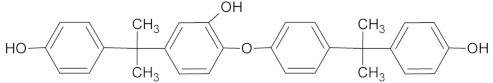
M4 *•	415	12.932	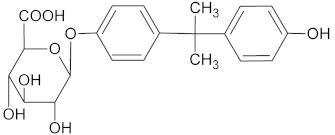
M5 •	243	8.846	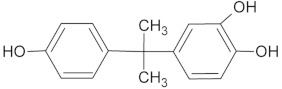

*—metabolite identified in cultures containing BPA; •*—*metabolite identified in cultures containing both BPA and copper sulfate; #*—*metabolite identified after incubation BPA solution with crude laccase of *M. roridum*.

**Table 2:** The effect of BPA on *M. roridum* IM 6482 membrane permeability (expressed as propidium iodide fluorescence in fungal biomass), lipid peroxidation (expressed as TBARS level) and ROS ratio (%) after 24 and 72 h of culturing in Czapek-Dox III medium containing 50 mg·L^−1^ of BPA. Asterisks represent values with statistically significant differences determined with a one-way ANOVA followed by the Tukey test (α = 0.05) for different treatments.

	Time of Cultivation (h)			
Parameter	24		72	
	Control	BPA	Control	BPA
**Propidium iodide fluorescence intensity (U·mg^−1^)**	3.12 ± 0.55	25.52 ± 4.02 *	17.19 ± 0.15	24.84 ± 1.36 *
**TBARS level (µM·g^−1^)**	1.43 ± 0.36	4.02 ± 0,09 *	1.42 ± 0.37	1.45 ± 0.04
**ROS ratio (%)**	0.27 ± 0.07	38.12 ± 6.53 *	0.53 ± 0.21	3.26 ± 1.24 *

## Data Availability

The data presented in this study are available on request from the corresponding author.

## Supplementary Materials

The following are available online at https://www.mdpi.com/article/10.3390/ijms221910676/s1
