# Supplementary material for: Bisphenol A Removal by the Fungus Myrothecium roridumIM 6482—Analysis of the Cellular and Subcellular Level
Source: Int J Mol Sci. 2021 Oct 1;22(19):10676. doi: 10.3390/ijms221910676 (PMC8509184; doi:10.3390/ijms221910676)
Supplement: Supplementary file 1 [file ijms-22-10676-s001.zip › ijms-1387906-supplementary.pdf]

A

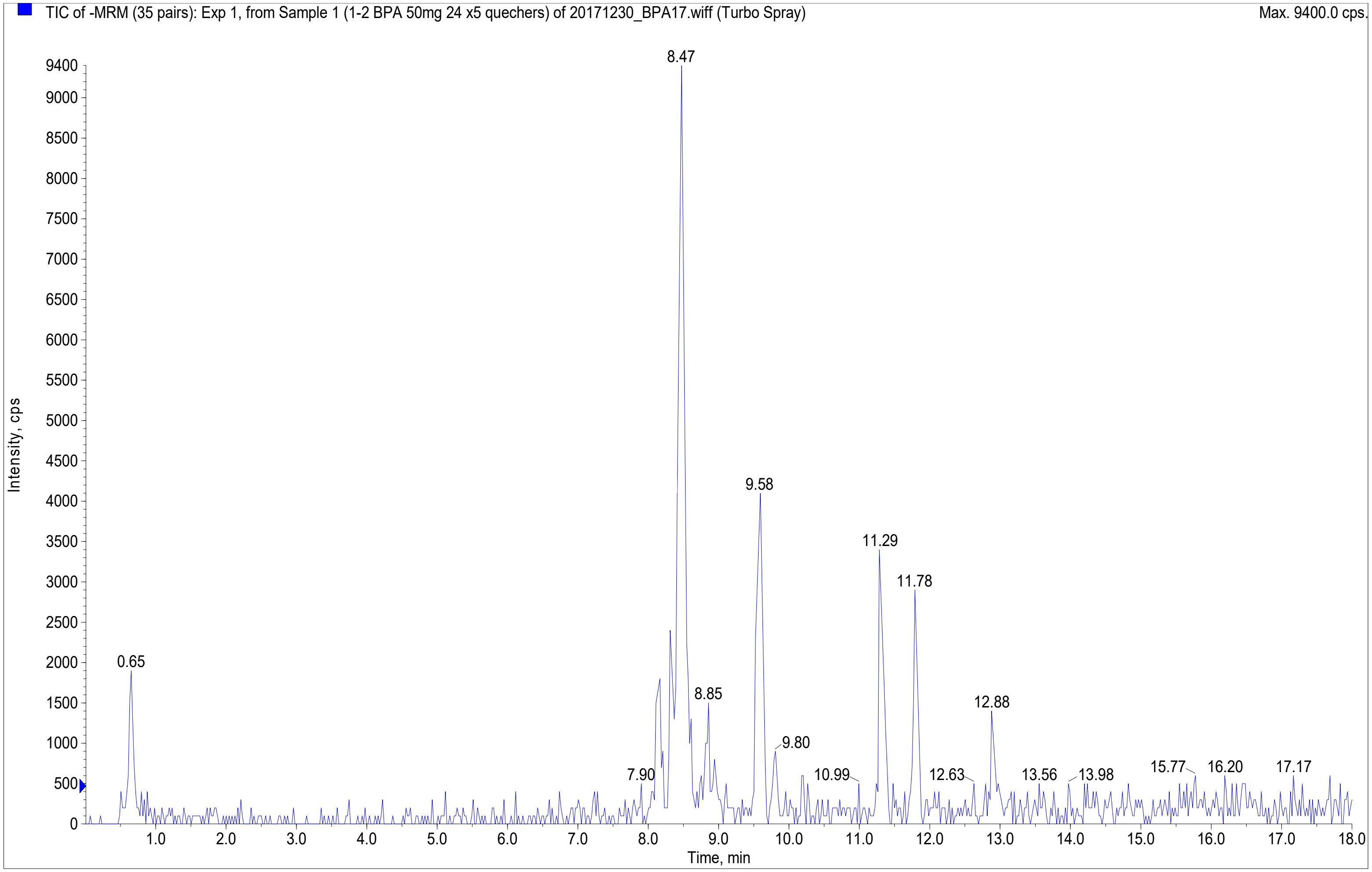
TIC of -MRM (35 pairs): Exp 1, from Sample 1 (1-2 BPA 50mg 24 x5 quechers) of 20171230_BPA17.wiff (Turbo Spray)
Max. 9400.0 cps.
Intensity, cps
Time, min
8.47
9.58
11.29
11.78
0.65
8.85
12.88
9.80
7.90
10.99
12.63
13.56
13.98
15.77
16.20
17.17

B

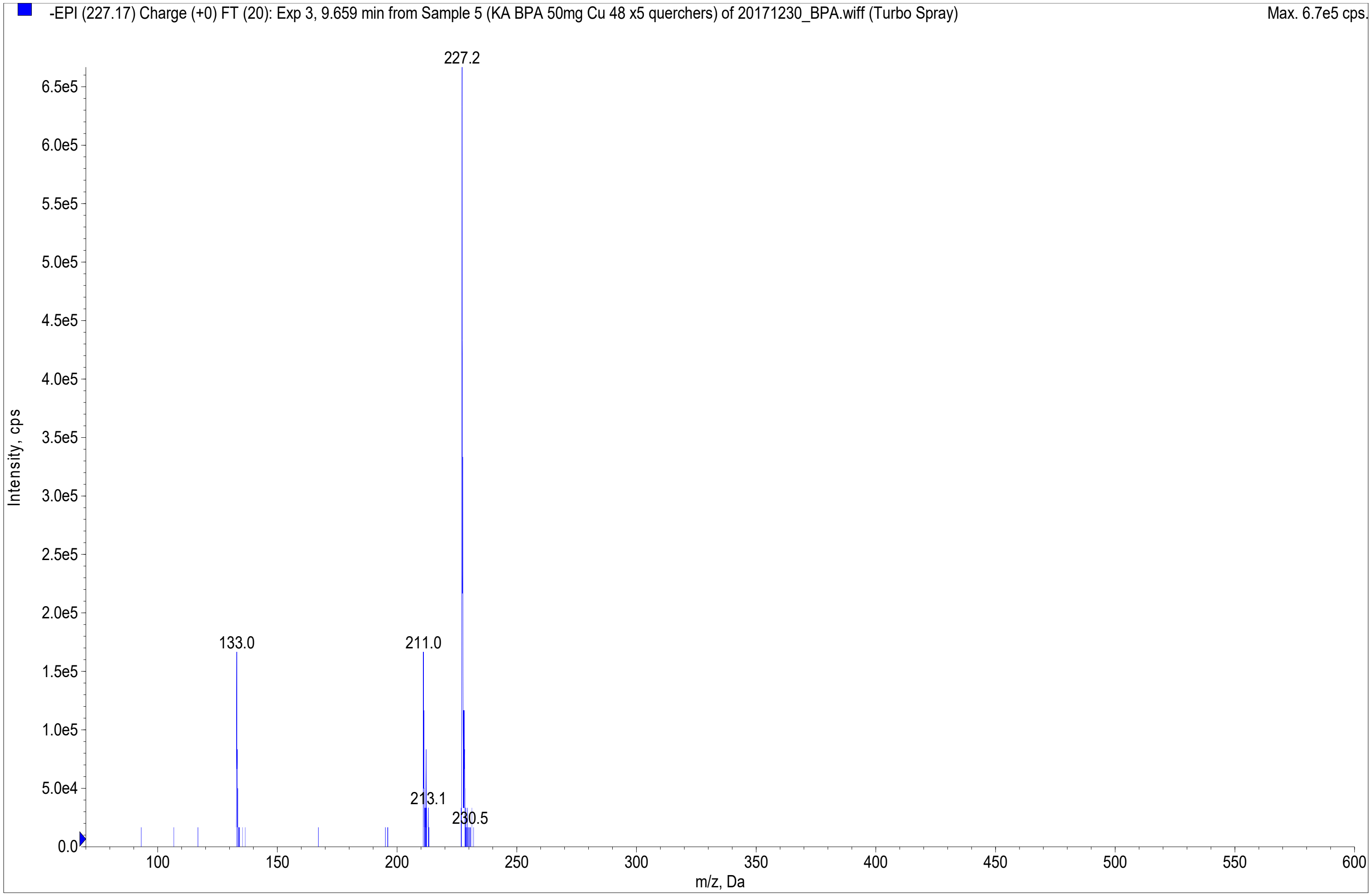
-EPI (227.17) Charge (+0) FT (20): Exp 3, 9.659 min from Sample 5 (KA BPA 50mg Cu 48 x5 querchers) of 20171230_BPA.wiff (Turbo Spray)
Max. 6.7e5 cps.
227.2
133.0
211.0
213.1
230.5
Intensity, cps
6.5e5
6.0e5
5.5e5
5.0e5
4.5e5
4.0e5
3.5e5
3.0e5
2.5e5
2.0e5
1.5e5
1.0e5
5.0e4
0.0
100
150
200
250
300
350
400
450
500
550
600
m/z, Da

c

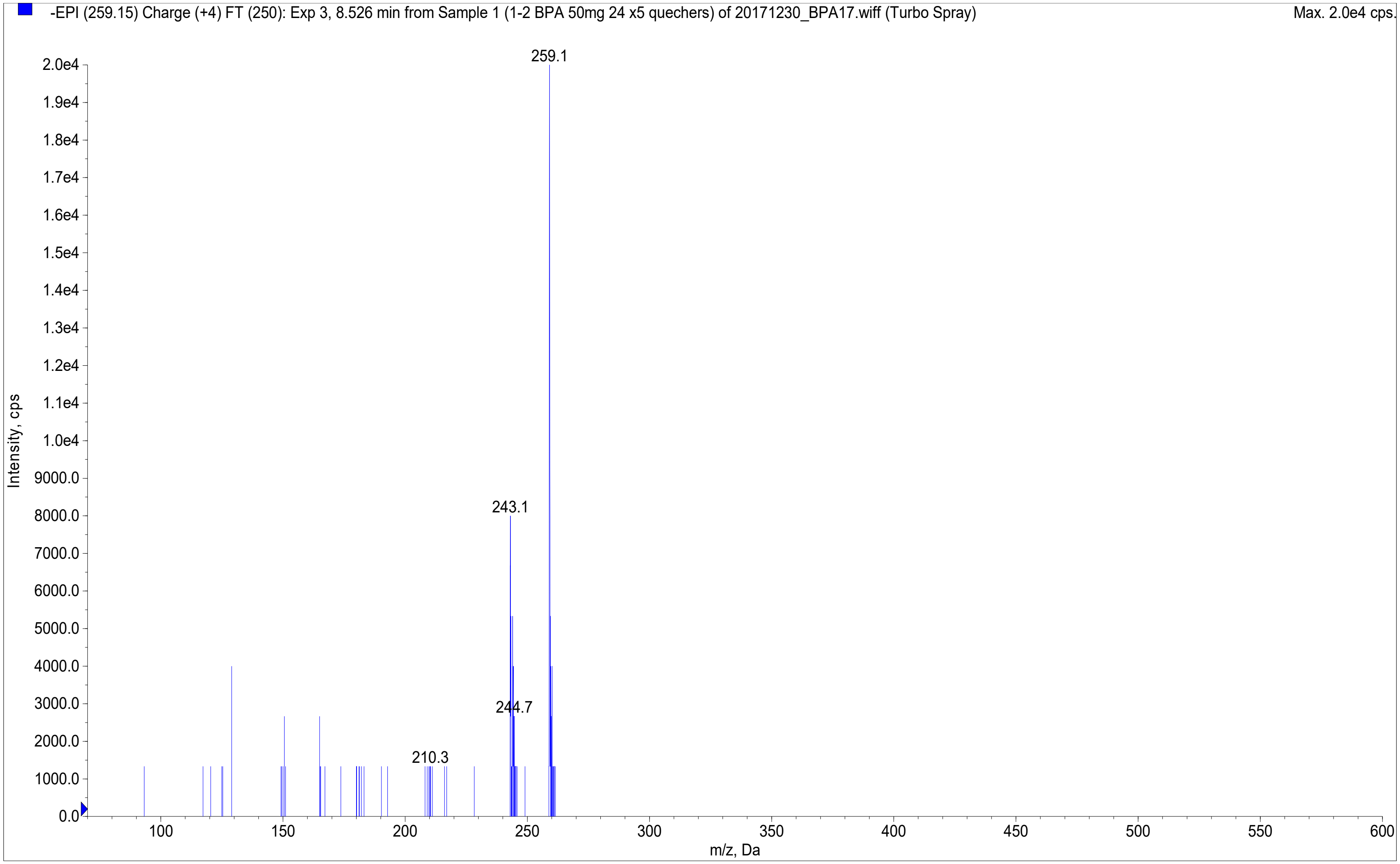

-EPI (259.15) Charge (+4) FT (250): Exp 3, 8.526 min from Sample 1 (1-2 BPA 50mg 24 x5 quechers) of 20171230_BPA17.wiff (Turbo Spray)
Max. 2.0e4 cps.
Intensity, cps
m/z, Da
259.1
243.1
244.7
210.3
2.0e4
1.9e4
1.8e4
1.7e4
1.6e4
1.5e4
1.4e4
1.3e4
1.2e4
1.1e4
1.0e4
9000.0
8000.0
7000.0
6000.0
5000.0
4000.0
3000.0
2000.0
1000.0
0.0
100
150
200
250
300
350
400
450
500
550
600

D

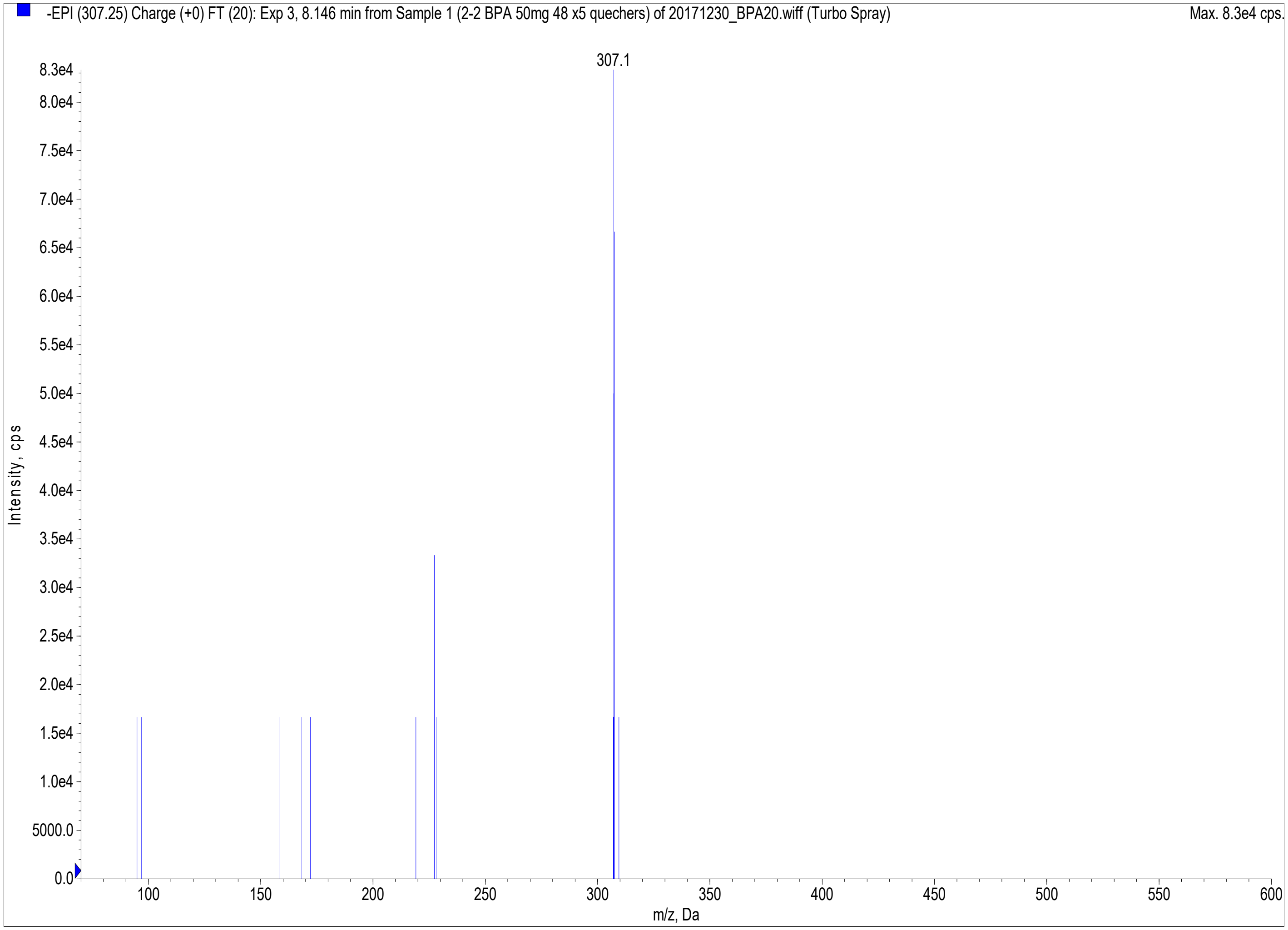

-EPI (307.25) Charge (+0) FT (20): Exp 3, 8.146 min from Sample 1 (2-2 BPA 50mg 48 x5 quechers) of 20171230_BPA20.wiff (Turbo Spray)
Max. 8.3e4 cps.
307.1
Intensity, cps
8.3e4
8.0e4
7.5e4
7.0e4
6.5e4
6.0e4
5.5e4
5.0e4
4.5e4
4.0e4
3.5e4
3.0e4
2.5e4
2.0e4
1.5e4
1.0e4
5000.0
0.0
100
150
200
250
300
350
400
450
500
550
600
m/z, Da

E

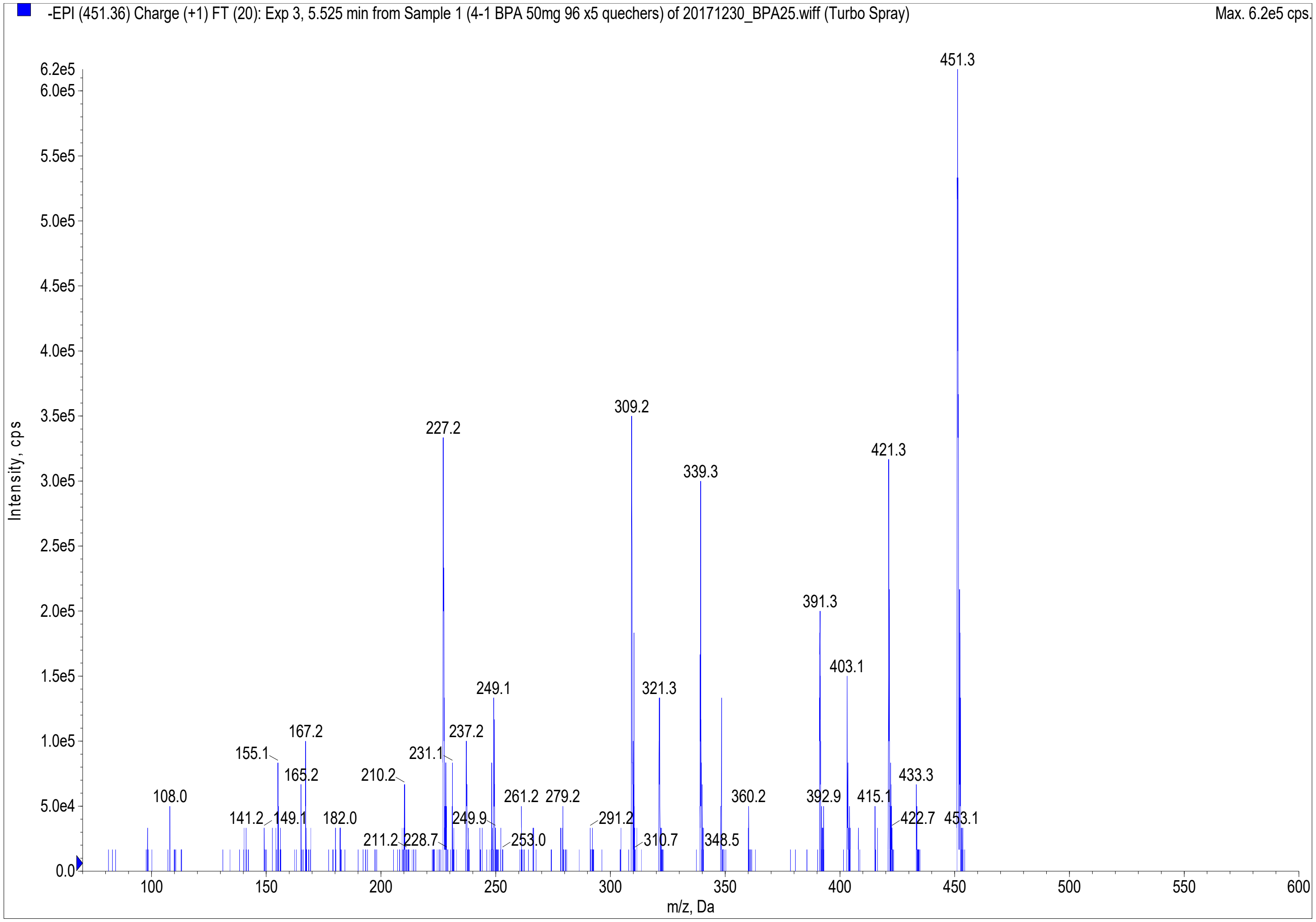
-EPI (451.36) Charge (+1) FT (20): Exp 3, 5.525 min from Sample 1 (4-1 BPA 50mg 96 x5 quechers) of 20171230_BPA25.wiff (Turbo Spray)
Max. 6.2e5 cps.
Intensity, cps
m/z, Da
451.3
309.2
227.2
339.3
421.3
391.3
403.1
249.1
321.3
167.2
237.2
155.1
231.1
165.2
210.2
108.0
261.2
279.2
360.2
392.9
415.1
433.3
141.2
149.1
182.0
249.9
291.2
422.7
453.1
211.2
228.7
253.0
310.7
348.5

F

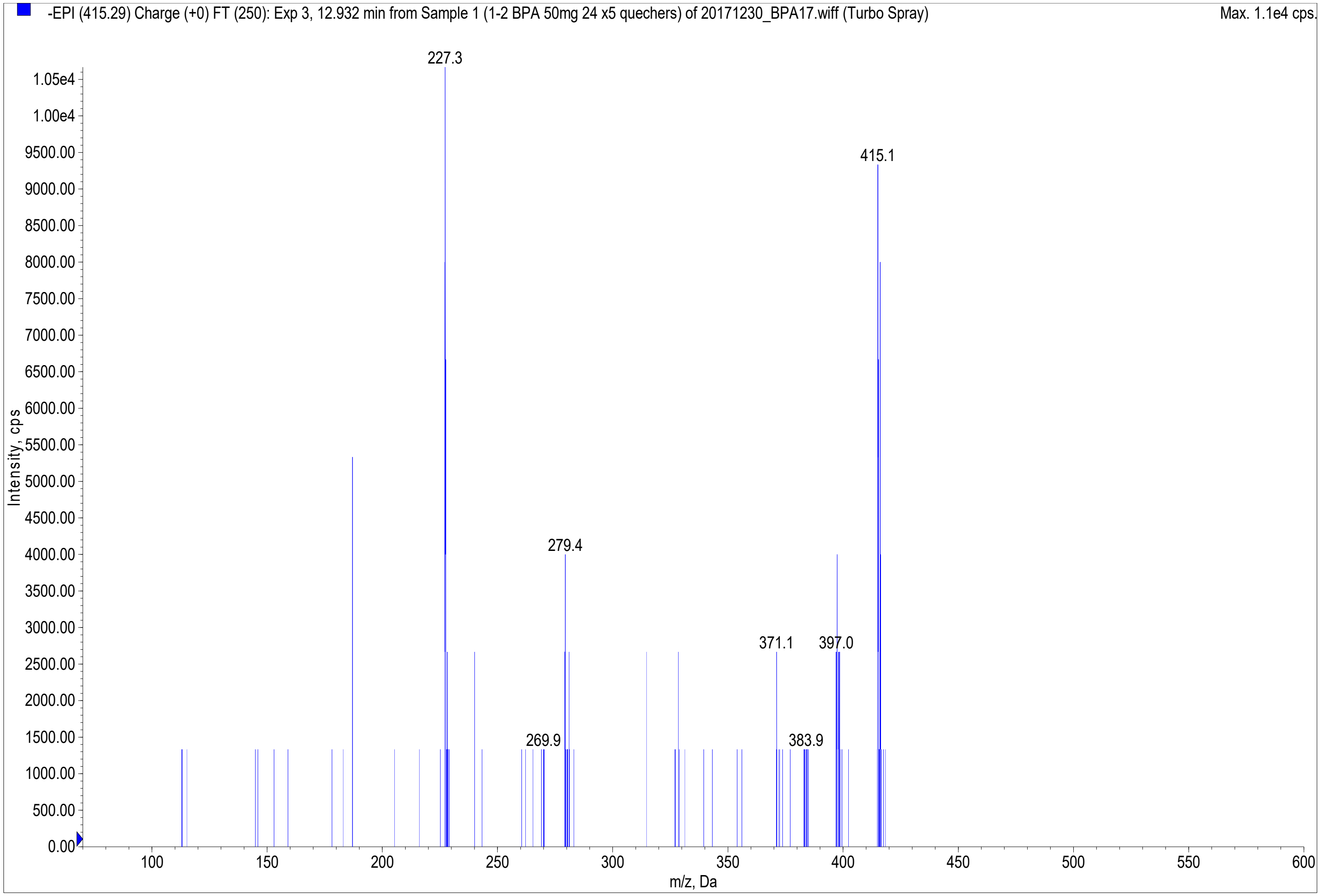

-EPI (415.29) Charge (+0) FT (250): Exp 3, 12.932 min from Sample 1 (1-2 BPA 50mg 24 x5 quechers) of 20171230_BPA17.wiff (Turbo Spray)
Max. 1.1e4 cps.
227.3
415.1
279.4
371.1
397.0
269.9
383.9
Intensity, cps
1.05e4
1.00e4
9500.00
9000.00
8500.00
8000.00
7500.00
7000.00
6500.00
6000.00
5500.00
5000.00
4500.00
4000.00
3500.00
3000.00
2500.00
2000.00
1500.00
1000.00
500.00
0.00
100
150
200
250
300
350
400
450
500
550
600
m/z, Da

G

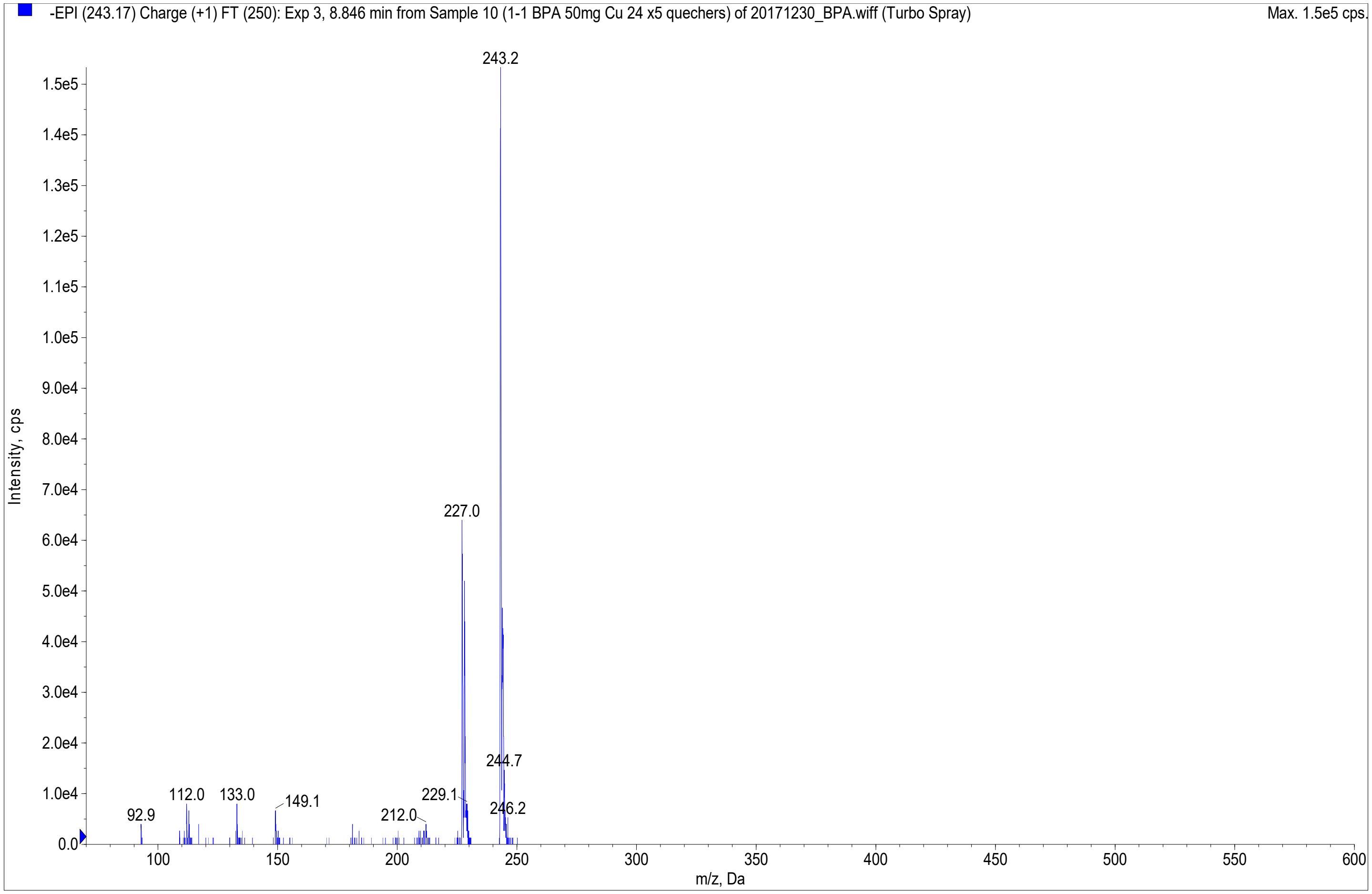

Figure S1. Chromatogram obtained after extraction of *M. roridum* cultures cultivated in Czapek-Dox medium supplemented with 50 mg l^{-1} BPA (A) and mass spectra of BPA (B) and its metabolites: M1 (C), M2 (D), M3 (E), M4 (F), M5 (G) formed in fungal cultures.

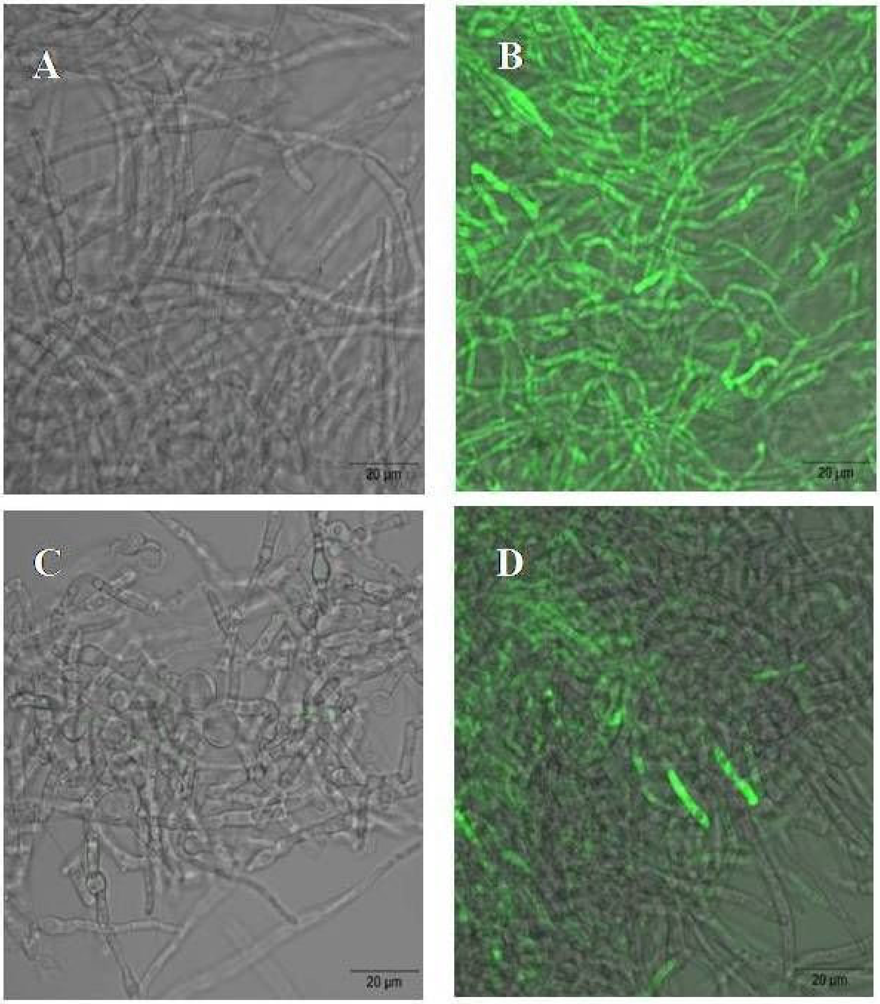

Figure S2. ROS generation in the *M. roridum* cultures. Control without BPA after 24 h (A) and 72 h (C); cultures supplemented with BPA after 24 h (B) and 72 h (D). The hyphal fluorescence indicates ROS generation.

Tabela S1. Summary identification of *M. roridum IM 6482* proteins isolated from control cultures (without BPA).

Spot no.	Best Protein Accession	Best Protein Mass	Best Protein Score	Best Protein Description	Organism
2	PQK16867.1	11454	148	hypothetical protein BB8028_0007g00680	*Beauveriabassiana*
5	OAA81287.1	19600	121	peptidyl-prolyl cis-trans isomerase	*Cordycepsconfragosa* RCEF 1005
10	EEU44844.1	16912	91	predicted protein	*Nectriahaematococca*mpVI 77-13-4
19	PTD04586.1	28552	79	40S ribosomal protein S3	*Fusariumculmorum*
22	XP_018656864.2	34018	199	malatedehydrogenase, partial	*Trichodermagamsii*
29	XP_018180540.1	59623	92	ATP synthase alpha chain, mitochondrial precursor	*Purpureocilliumlilacinum*
32	PTB35940.1	78531	84	hypothetical protein M441DRAFT_152105	*Trichodermaasperellum*CBS 433.97
55	KOS18933.1	54440	155	ATP synthasesubunit beta	*Escovopsisweberi*
57	XP_016588082.1	69320	103	heatshock 70kDa protein 1/8	*Sporothrixschenckii* 1099-18
59	PTB80850.1	47299	80	phosphopyruvatehydratase	*Trichodermalongibrachiatum* ATCC 18648 0.1
60	ODA79096.1	42721	95	hypothetical protein RJ55_04687	*Drechmeriaconiospora*

Spot no.	Best Protein Accession	Best Protein Mass	Best Protein Score	Best Protein Description	Organism
66	KIL95357.1	77769	155	hypothetical protein FAVG1_00093	*Fusariumavenaceum*
67	GAO16225.1	72752	103	hypothetical protein UVI_02000080	*Ustilaginoideavirens*
68	PTB80850.1	47299	155	phosphopyruvatehydratase	*Trichodermalongibrachiatum* ATCC 18648
69	PTB80850.1	47299	146	phosphopyruvatehydratase	*Trichodermalongibrachiatum*ATCC 18648
70	PTB80850.1	47299	138	phosphopyruvatehydratase	*Trichodermalongibrachiatum* ATCC 18648
71	ODA77078.1	26963	100	hypothetical protein RJ55_07596	*Drechmeriaconiospora*
74	POR33248.1	62028	97	Inorganicpyrophosphatase	*Tolypocladiumparadoxum*
78	XP_018139774.1	31537	174	coatomersubunit epsilon	*Pochoniachlamydosporia 170*
81	ORY66301.1	43858	128	S-adenosylmethioninesynthetase	*Pseudomassariellavexata*
86	PTB35940.1	78531	78	hypothetical protein M441DRAFT_152105	*Trichodermaasperellum* CBS 433.97
88	AID48697.1	37452	200	actin, partial	*Neotyphodium sp.* NI_201306

Spot no.	Best Protein Accession	Best Protein Mass	Best Protein Score	Best Protein Description	Organism
90	PQK13047.1	30402	84	hypothetical protein BB8028_0003g16610	*Beauveriabassiana*
91	POR33248.1	62028	98	Inorganicpyrophosphatase	*Tolypocladiumparadoxum*
92	OTA68683.1	25460	149	mitochondrialperoxiredoxin PRX1	*Hypoxylon sp.* EC38
96	XP_009260797.1	17927	119	hypothetical protein FPSE_09405	*Fusariumpseudograminearum*CS3096
97	PHH82764.1	18833	78	hypothetical protein CDD82_4929	*Ophiocordycepsaustralis*
98	PNY28828.1	26923	125	Actin cross-linking	*Tolypocladiumcapitatum*
99	PNY25025.1	13598	201	Profilin	*Tolypocladiumcapitatum*
100	XP_018160338.1	17955	156	Cytochrome c oxidase subunit Va	*Colletotrichumhigginsianum* IMI 349063
101	CCE28255.1	16874	90	related to cofilin	*Clavicepspurpurea* 20.1
105	EQL03272.1	36308	110	outer mitochondrial membrane protein porin	*Ophiocordycepssinensis*CO18
106	PTD05987.1	16456	112	Nascent polypeptide-associated complex subunit beta	*Fusariumculmorum*
107	KIL96612.1	10977	75	hypothetical protein FAVG1_01356	*Fusariumavenaceum*

Spot no.	Best Protein Accession	Best Protein Mass	Best Protein Score	Best Protein Description	Organism
108	AGY49501.1	12583	137	calmodulin, partial	*Fusariumandiyazi*
109	PTD11202.1	31749	78	14-3-3 protein	*Fusariumculmorum*
110	PNY29201.1	10793	117	60S acidic ribosomal protein P2	*Tolypocladiumcapitatum*
111	PTB69803.1	30245	96	14-3-3-like protein	*Trichodermacitrinoviride*
112	PTB69803.1	30245	96	14-3-3-like protein	*Trichodermacitrinoviride*

Tabela S2. Summary identification of *M. roridum IM 6482* proteins isolated from cultures containing 50 mg L^{-1} BPA.

Spot no.	Best Protein Accession	Best Protein Mass	Best Protein Score	Best Protein Description	Organism
115	PQK15291.1	22856	144	hypothetical protein BB8028_0005g08050	*Beauveriabassiana*
116	ABQ42572.1	36234	224	glyceraldehyde-3-phosphate dehydrogenase	*Myrotheciumgramineum*
117	PSR79323.1	11114	136	peptidyl-prolyl cis-trans isomerase fkr-3	*Coniellalustricola*
118	EEU44844.1	16912	173	predicted protein	*Nectriahaematococca*mpVI 77-13-4
119	EEU44844.1	16912	150	predicted protein	*Nectriahaematococca*mpVI 77-13-4
120	OAA81287.1	19600	284	peptidyl-prolyl cis-trans isomerase	*Cordycepsconfragosa*RCEF 1005
121	EEU44844.1	16912	115	predicted protein	*Nectriahaematococca*mpVI 77-13-4
122	PQK16867.1	11454	91	hypothetical protein BB8028_0007g00680	*Beauveriabassiana*
123	EEU45443.1	37541	475	predicted protein	*Nectriahaematococca*mpVI 77-13-4
124	KFH42761.1	49928	30	Elongation factor 1-alpha-like protein	*Acremoniumchrysogenum* ATCC 11550
125	PQK15291.1	22856	128	hypothetical protein BB8028_0005g08050	*Beauveriabassiana*

Spot no.	Best Protein Accession	Best Protein Mass	Best Protein Score	Best Protein Description	Organism
131	EEU42998.1	23091	131	predicted protein	*Nectriahaematococca*mpVI 77-13-4
133	POR36939.1	15791	91	Superoxidedismutase [Cu-Zn]	*Tolypocladiumparadoxum*
142	PNY25025.1	13598	79	Profilin	*Tolypocladiumcapitatum*
144	XP_018177206.1	36217	219	glyceraldehyde-3-phosphate dehydrogenase	*Purpureocilliumlilacinum*
145	XP_024404531.1	52336	130	adenylyl-sulfatekinase	*Trichodermagamsii*
146	POR37810.1	55242	322	ATP synthasesubunit beta	*Tolypocladiumparadoxum*
147	POR37810.1	55242	262	ATP synthasesubunit beta	*Tolypocladiumparadoxum*
148	POR35368.1	52948	344	Enolase, partial	*Tolypocladiumparadoxum*
149	EMT70814.1	35277	407	Malatedehydrogenase, mitochondrial	*Fusarium oxysporum f. sp. cubense* race 4
150	OAR02581.1	38327	509	hypothetical protein LLEC1_00332, partial	*Cordycepsconfragosa*
151	KZZ90744.1	34989	521	malatedehydrogenaseprecursor	*Moelleriella libera* RCEF 2490
152	XP_018136472.1	40886	159	acetyl-CoA C-acetyltransferase	*Pochoniachlamydosporia* 170

Spot no.	Best Protein Accession	Best Protein Mass	Best Protein Score	Best Protein Description	Organism
153	XP_022284686.1	58260	419	6-phosphogluconate dehydrogenase	*Pochoniachlamydosporia* 170
154	EEU44203.1	77244	85	hypothetical protein NECHADRAFT_65315	*Nectriahaematococca*mpVI 77-13-4
155	KIL87093.1	81262	202	catalase-peroxidase	*Fusariumavenaceum*
156	EFY93483.1	73112	508	heat shock 70 kDa protein precursor	*Metarhiziumacridum*CQMa 102
157	PQK16496.1	PQK16496.1	141	hypothetical protein BB8028_0006g08160	*Beauveriabassiana*
158	ABQ42572.1	ABQ42572.1	548	glyceraldehyde-3-phosphate dehydrogenase	*Myrotheciumgramineum*
159	XP_013944143.1	36069	284	UDP-glucose 4-epimerase Gal10	*Trichodermaatroviride* IMI 206040
162	ǀQCR2_NEUCR	40553	75	Ubiquinol-cytochrome-c reductase complex core protein	*Neurosporacrassa*OR74A
163	GAO18194.1	36533	452	hypothetical protein UVI_02036930	*Ustilaginoideavirens*
164	KFA79660.1	46953	380	hypothetical protein S40288_04082	*Stachybotryschartarum* IBT 40288
165	XP_018235168.1	19005	178	tyrosine 3-monooxygenase/tryptophan 5-monooxygenase activation protein	*Fusariumoxysporum f. sp. lycopersici*4287
166	KFA60529.1	56642	101	hypothetical protein S40285_07587	*Stachybotryschlorohalonata*IBT 40285

Spot no.	Best Protein Accession	Best Protein Mass	Best Protein Score	Best Protein Description	Organism
167	PQK16496.1	36069	126	hypothetical protein BB8028_0006g08160	*Beauveriabassiana*
168	PTB77983.1	24242	106	HSP20-like chaperone	*Trichodermalongibrachiatum* ATCC 18648
169	KFA78328.1	79469	239	hypothetical protein S40288_05032	*Stachybotryschartarum* IBT 40288
170	KFA79660.1	47482	430	hypothetical protein S40288_04082	*Stachybotryschartarum* IBT 40288
171	KZZ94886.1	65977	1050	ATP synthase beta chain precursor	*Moelleriella libera* RCEF 2490]
172	KND89290.1	42624	170	Vacuolarprotease A	*Tolypocladiumophioglossoides* CBS 100239
173	PQK09468.1	55548	156	hypothetical protein BB8028_0001g1538	*Beauveriabassiana*
174	XP_018655921.1	59487	146	ATP synthase subunit alpha, mitochondrial	*Trichodermagamsi*
175	XP_018658099.1	31900	212	40S ribosomal protein S0	*Trichodermagamsii*
176	XP_018656673.1	54863	254	ATP synthasesubunit beta	*Trichodermagamsii*
178	PTB36680.1	38397	142	hypothetical protein M441DRAFT_176232	*Trichodermaasperellum* CBS 433.97
179	AQX77513.1	40124	437	actin gamma, partial	*Gliocladium sp.*

Spot no.	Best Protein Accession	Best Protein Mass	Best Protein Score	Best Protein Description	Organism
180	CCE27930.1	30012	248	probable 14-3-3-like protein	*Clavicepspurpurea* 20.1
181	KFA76311.1	17815	339	hypothetical protein S40288_02977	*Stachybotryschartarum* IBT 40288
182	CEI40789.1	72567	81	unnamed protein product	*Fusariumvenenatum]*
183	XP_018183267.1	17532	225	translationelongationfactor, IF5A	*Purpureocilliumlilacinum*
184	KFA79660.1	47482	241	hypothetical protein S40288_04082	*Stachybotryschartarum* IBT 40288
185	XP_018656887.1	23353	236	hypothetical protein TGAM01_v209462	*Trichodermagamsii*
186	OAA44345.1	141716	99	1,4-alpha-glucan branchingenzyme	*Metarhiziumrileyi* RCEF 4871
189	KDB12143.1	33116	109	proteasome regulatory subunit 12	Ustilaginoideavirens
190	AQX77513.1	40124	437	actin gamma, partial	Gliocladium sp.
191	EFY92780.1	45159	246	TH14-3-3 like protein	MetarhiziumacridumCQMa 102
192	KIL96612.1	10977	82	hypothetical protein FAVG1_01356	*Fusariumavenaceum*
194	XP_018160338.1	17955	346	Cytochrome c oxidase subunit Va	*Colletotrichumhigginsianum* IMI 349063
195	PQK16496.1	36069	98	hypothetical protein BB8028_0006g08160	*Beauveriabassiana*

Spot no.	Best Protein Accession	Best Protein Mass	Best Protein Score	Best Protein Description	Organism
196	XP_018181948.1	27661	149	RanBP1 domain-containing protein	*Purpureocilliumlilacinum*
197	KFH44584.1	36904	114	hypothetical protein ACRE_046720	*Acremoniumchrysogenum* ATCC 11550
198	XP_023424735.1	70964	212	probable heat shock protein 70 (hsp70)	*Fusariumfujikuroi* IMI 58289]
199	EJP67331.1	72459	177	hsp70-like protein	Beauveriabassiana ARSEF 2860
201	KLO87050.1	15126	98	putative ribosomal protein S19.e, cytosolic	*Fusariumfujikuroi*
203	AID48698.1	37472	88	actin, partial	*Neotyphodium sp*. NI_201308]
